# Sero‐epidemiology investigation of *Coxiella burnetii* in domestic ruminants throughout most Greek regions

**DOI:** 10.1002/vms3.337

**Published:** 2020-08-12

**Authors:** Dimitrios Vourvidis, Anna Kyrma, Maria Linou, Sophie Edouard, Emmanouil Angelakis

**Affiliations:** ^1^ Ministry of Rural Development and Food Attica Greece; ^2^ Laboratory of Medical Microbiology Hellenic Pasteur Institute Athens Greece; ^3^ Aix Marseille Univ IRD IHU Méditerranée Infection MEPHI Marseille France; ^4^ Aix Marseille Université, IRD APHM VITROME IHU‐Méditerranée Infection Marseille France

**Keywords:** *Coxiella burnetii*, Greece, livestock, Q fever, surveillance

## Abstract

Q fever is not considered as a public health problem in Greece where most regions are considered as *Coxiella burnetii* free possibly because of the low interest for this agent. Our objective was to conduct a large‐scale study to investigate the sero‐epidemiology of *C. burnetii* in domestic ruminants throughout the most of Greek regions. We tested serum samples obtained from goats, sheep and bovines from different regions of Greece. All sera were tested for *C. burnetii* IgG antibodies by a commercial ELISA according to the manufacturer's recommendations. We tested 1,173 goats and sheep obtained from 177 different herds and totally 194 (17%) animals from 78 (44%) herds were positive for *C. burnetii*. Positive animals were present in seven (88%) different regions and seropositivity varied widely among these regions. The highest percentage was observed in Peloponnese (44%), where all the tested herds presented animals with *C. burnetii* antibodies. Ιn all Aegean Islands except the island of Limnos we detected goats and sheep positive for *C. burnetii* with seroposivity varying between 2% in Kos to 37% in Rhodes. Finally, in 22 (85%) Greek prefectures we found *C. burnetii* IgG‐positive animals whereas in 14 (54%) prefectures more than 50% of tested herds had seropositive animals. We also tested 28 cows from five different herds in Macedonia and Aegean Islands and six (21%) of them, obtained from two (40%) herds were positive. Considering the importance of *C. burnetii* for public health, our data reflect the lack of awareness by veterinarians, physicians and competent authorities as we provide evidence of *C. burnetii* seropositivity in productive animals throughout the most of Greek territories. Due to the increased risk of inhalation of the bacterium by people who entered the affected farms we raise the question of Q fever emergence in Greece.

## Graphical abstract

Q fever is not considered as a public health problem in Greece and few human cases have been recorded possibly because of the low interest for this agent. We conducted a large‐scale study to investigate the sero‐epidemiology of *C*. *burnetii* in domestic ruminants and we provide evidence of *C*. *burnetii* presence in productive animals throughout the most of Greek regions.

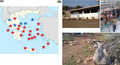

## 1 INTRODUCTION

Q fever is a worldwide zoonosis caused by an obligate intracellular bacterium, *Coxiella burnetii* (Angelakis & Raoult, 2010). In human, clinical findings in Q fever infection are often confusing, and primary infection is asymptomatic in approximately 60% of cases. Cardiovascular complications are the main risk of *C burnetii* infection, including acute and chronic endocarditis and vascular infections (Million et al., 2016; Million, Walter, Thuny, Habib, & Raoult, 2013). Indeed, Q fever endocarditis is associated with surgery for 15% to 73% of patients, causes death in 5% to 65% of patients, and induces a large number of relapses when it is inadequately treated (Prudent, Lepidi, Angelakis, & Raoult, 2018). *C. burnetii* could also cause obstetric complications including abortion or foetal malformations in pregnant women (Angelakis et al., 2012).

The main reservoirs of *C. burnetii* are cattle, sheep, and goats. In most cases, human contamination occurs from inhalation of aerosolized bacteria that are spread in the environment from animal birth products (Angelakis & Raoult, 2010) and findings suggest the role of wind in the transmission of *C. burnetii* between ruminants and humans (Pandit, Hoch, Ezanno, Beaudeau, & Vergu, 2016; Tissot‐Dupont, Amadei, Nezri, & Raoult, 2004). Moreover, introduction of new animals into herds has been identified as a risk factor of *C. burnetii* infection and it is known that trade between cattle herds occurs frequently and sometimes over long distances (Nusinovici et al., 2014). In livestock, infections caused by *C. burnetii* are usually asymptomatic although the disease has been implicated in abortion, stillbirths, endometritis, mastitis and infertility (Arricau‐Bouvery & Rodolakis, 2005). Recently, there has been an increased awareness of Q fever as an economically important pathogen due to a rise in the frequency of reported outbreaks and the economic impact of Q fever has on commercial livestock operations in the form of lost animal reproductive productivity and herd death (Enserink, 2010). The importance of Q fever, in terms of public health, increased after the outbreak in the Netherlands, where more than 4,000 people became ill and 50,000 animals were slaughtered to control the epidemic (Van Der Hoek et al., 2012).

Although the classification of *C. burnetii* by the CDC as a potential bioterrorism agent resulted in the disease becoming reportable in many countries (Eldin et al., 2017), Q fever is not considered as a public health problem in Greece and few human cases have been recorded (Kokkini et al., 2013). However, we recently raised the question of the under‐diagnosis of human *C. burnetii* infections in Greece (Karageorgou et al., 2020). Previously, it was found that the *C. burnetii* genotype MST32 is circulating in sheep and goat at eight different areas of Northern Greece (Chochlakis et al., 2018). However, most Greek regions are still considered as Q fever free possibly because of the low interest for this agent and to the best of our knowledge only two sero‐epidemiological studies have been previously conducted to estimate *C. burnetii* in domestic ruminants in Greece (Filioussis et al., 2017; Pape et al., 2009). In association with the Greek Ministry of Rural Development and Food, we conducted a large‐scale pilot study throughout the most of Greek regions to determine for the first time if Q fever is a concern in domestic ruminants in Greece.

## 2 MATERIALS

### 2.1 Sample collection

From January 2015 to December 2019 we tested serum samples, following communication with local veterinarians, obtained from goats, sheep and bovines from different regions of Greece. The participation to our study was voluntary and we encouraged veterinarians to sample animals with a clinical diagnosis of any of the following adverse pregnancy outcomes: abortion, premature delivery, stillbirth and weak offspring. In order to facilitate the participation and increase the number of tested farms, we did not force veterinarians to collect other than the serum samples information. Serum samples were collected from each animal suspected for Q fever and within 6 hr of collection were sent to our laboratory stored at −20°C for further analysis.

### 2.2 Ethics statement

Animal sampling were conducted according to the guidelines of the Greek National Veterinary Agency.

### 2.3 Serology assays

The serum antibody IgG titres against *C. burnetii* were assayed using a Q fever indirect ELISA kit (IDEXX Q‐Fever (*C. burnetii*) Antibody Test, IDEXX Laboratories, Inc), with a mixture of *C. burnetii* phases I and II antigen according to the manufacturer's instructions. The manufacturer had previously validated the ELISA kit estimating sensitivity of 100% and specificity of 95%, respectively. Briefly, the serum samples were incubated at room temperature for 30 min and then diluted in a 1:20 sample diluent. Negative and positive controls were always included while examining serum samples. ELISA results were obtained by comparing the optical density (OD) of the sample well with the OD from the positive control. For each sample we calculated the ELISA index S/P % according to the manufacturer's instructions using a photometer at a wavelength of 450 nm as follows: S/P % = 100 × (OD value of the sample tested − OD value of the negative control)/ (OD of the positive control − OD of the negative control). Sera samples with S/P % <30% were considered negative, whereas samples with S/P % ≥40 were considered positive. A sample with 30% ≤SP % <40% was considered suspect and reanalysed again.

### 2.4 Statistical analysis

Student *t* or *χ* ^2^ tests were performed using Epi Info version 6.0 software (Centers for Disease Control and Prevention, Atlanta, GA, USA). Means were compared using analysis of variance or the Kruskal–Wallis test, on the basis of results of the Bartlett test for inequality of population variances. Proportions were compared using the Mantel–Haenszel *χ* ^2^ or Fisher exact tests when the expected value of a cell was <0.05. A *p* value < .05 was considered significant.

## 3 RESULTS

**FIGURE 1:**
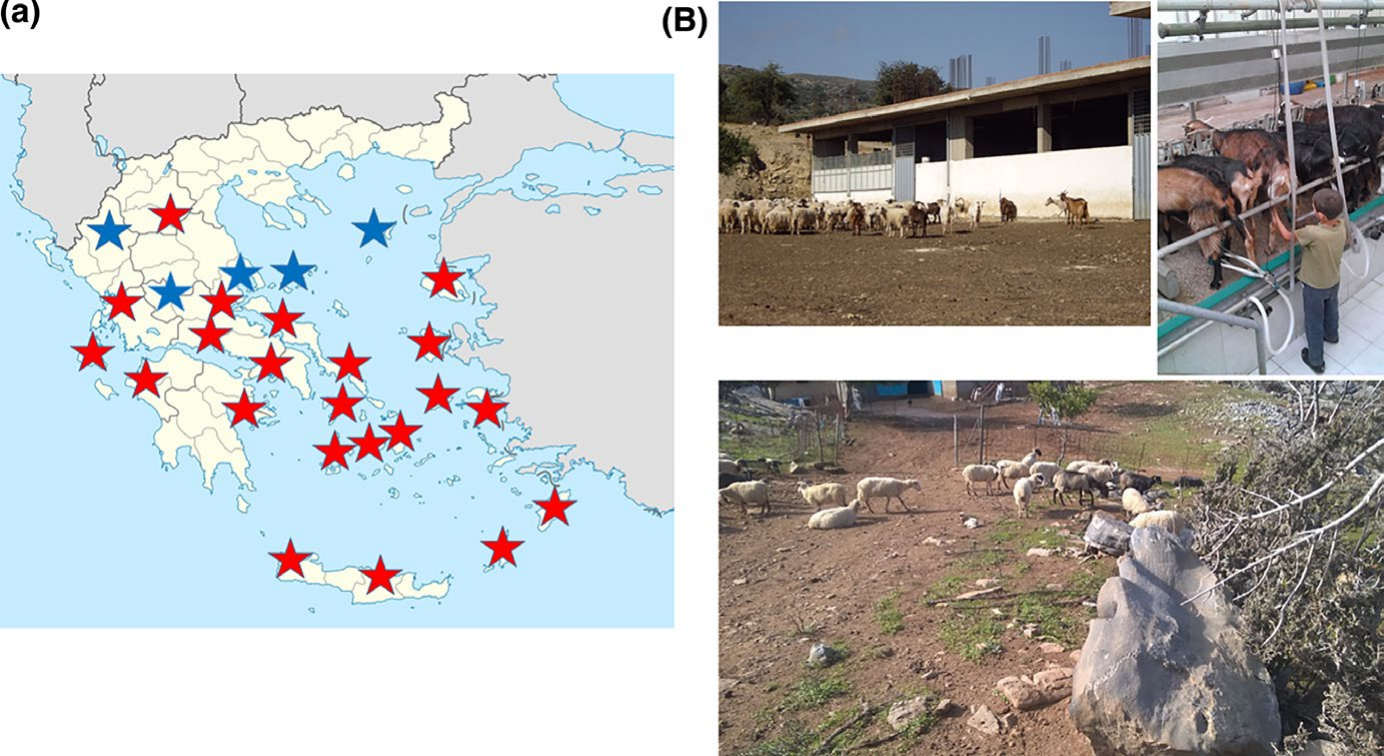
(a) Greek prefectures tested for *Coxiella burnetii* seropositivity. (b) Goat and sheep pastoral herds in Greece. Blue stars: prefectures without *C. burnetii* seropositive animals, red stars: prefectures with *C. burnetii* sero‐positive animals

We tested 1,173 goats and sheep obtained from 177 different herds located in eight out of the nine geographic regions of Greece (Figure 1a). Overall, 194 (17%) animals from 78 (44%) herds were seropositive for *C. burnetii* (Table 1). Positive animals were present in seven (88%) regions and seropositivity varied widely among these regions. Indeed, the highest percentage of *C. burnetii* seropositivity was observed in Peloponnese (44%), where all the tested herds had positive animals. However, this high seropositivity can be due to the small number of herds that we tested in Peloponnese. Surprisingly, in all Aegean Islands except the island of Limnos we detected goats and sheep with *C. burnetii* antibodies and seropositivity varied between 2% in Kos to 37% in Rhodes. Finally, in 22 (85%) Greek prefectures we found *C. burnetii* positive animals whereas in 14 (54%) prefectures more than 50% of tested herds had seropositive animals. The highest presence of *C. burnetii* antibodies was observed in the prefectures of Argolida (88%), followed by Rhodes (37%) and Lesbos (35%).

**TABLE 1:** Seropositivity of *Coxiella burnetii* in dairy sheep and goats in Greece

Regions	Prefectures	Tested herds	Positives herds (%)	Tested animals	Seropositive animals (%)
Macedonia	Grevena	8	2 (25%)	47	4 (9%)
Epirus	Ioannina	2	0 (0%)	34	0 (0%)
	Preveza	1	1 (100%)	3	1 (33%)
Central Greece	Attika	21	6 (29%)	58	9 (15%)
	Viotia	11	6 (55%)	111	8 (7%)
	Euritania	1	0 (0%)	7	0 (0%)
	Fthiotida	23	1 (4%)	56	1 (2%)
	Euboea island	16	8 (50%)	53	8 (15%)
Thessaly	Magnisia‐Sporades	1	0 (0%)	9	0 (0%)
Peloponnese	Argolida	1	1 (100%)	8	7 (88%)
	Ahaia	1	1 (100%)	17	4 (24%)
Aegean Islands	Andros	7	2 (29%)	11	2 (18%)
	Lesbos	12	10 (83%)	80	28 (35%)
	Limnos	1	0 (0%)	4	0 (0%)
	Naxos	25	14 (56%)	176	41 (23%)
	Rhodes	9	8 (89%)	27	10 (37%)
	Syros	4	3 (75%)	69	11 (16%)
	Myconos	1	1 (100%)	30	7 (23%)
	Kythnos	3	2 (67%)	47	7 (15%)
	Kos	5	1 (20%)	47	1 (2%)
	Leros	1	1 (100%)	17	6 (35%)
	Karpathos	1	1 (100%)	13	2 (15%)
	Samos	4	1 (25%)	73	3 (4%)
Ionian Islands	Kefalonia	7	1 (14%)	56	5 (9%)
Crete	Hania	6	5 (83%)	93	22 (24%)
	Iraklio	5	2 (40%)	27	7 (26%)
Total		177	78 (44%)	1,173	194 (17%)

Statistical comparison of seropositivity among the eight regions showed that significant more animals presented *C. burnetii* antibodies in Peloponnese comparing to all other regions (*p* < .05) where the probability of the presence of *C. burnetii* seropositive animals was 100% (*p* < .01). Moreover, significant more animals had *C. burnetii* antibodies in Aegean Islands comparing to Central Greece (*p* = .0001), Macedonia (*p* = .0001), Ionian Islands (*p* = .05) and Epirus (*p* = .005) whereas significant more animals were seropositive in Crete comparing to Macedonia (*p* = .03), Epirus (*p* = .003) and Ionian Islands (*p* = .02).

We also tested 28 cows from five different herds in Macedonia and Aegean Islands and six (21%) of them, obtained from two (40%) herds were positive (Table 2). We did not find significant difference in *C. burnetii* seropositivity between goats‐sheep and cows (*p* = .4). Similarly, we did not find difference between herds with goat and sheep versus bovine herds positives for *C. burnetii* (*p* = .9).

**TABLE 2:** Seropositivity of *C. burnetii* in bovines in Greece

Prefectures	Provinces	Tested herds	Positives herds (%)	Tested animals	Seropositives animals (%)
Macedonia	Grevena	2	0 (0%)	9	0 (0%)
Aegean Islands	Naxos	3	2 (67%)	19	6 (32%)
Total		5	2 (40%)	28	6 (21%)

## 4 DISCUSSION

We provide evidence of *C. burnetii* presence in productive animals throughout the most of Greek territories. Only two small‐scale studies have been previously performed in Greece to estimate the presence of *C. burnetii* antibodies in sheep and goats (Filioussis et al., 2017; Pape et al., 2009) and none in cattle. Although validated commercial ELISA kits are routinely used for the detection of *C. burnetii* in productive animals with high sensitivity and specificity, a limitation of our study is that we did not confirm our results by a specific immunofluorescence assay (IFA). Moreover, we did not perform molecular assays in positive samples to detect the *C. burnetii* genotypes that circulate in productive animals in Greece (Chochlakis et al., 2018). We believe that the determination of *C. burnetii* genotypes would be important as the clinical manifestations of Q fever in human depend, at least in part, on the *C. burnetii* genotype (Angelakis et al., 2012). Indeed, although acute clinical presentation is strain‐specific, all genotypes have been associated with endocarditis in human (Angelakis et al., 2012). Another limitation is that we could not discriminate the goat and sheep samples as their origin was from mixed goat and sheep herds and no species‐specific information was provided to identify the samples. Indeed, most of herds in Greece, continue to maintain traditional pastoral practices with mixed goat and sheep livestock (Figure 1b). Moreover, information about animal level and herd level demographic characteristics, livestock rearing system were not collected due to the reluctance of veterinary volunteers as was significantly increased their work.

We found that seropositivity of *C. burnetti* in the different prefectures of Greece is very variable from one area to another. Similar regional differences in the prevalence of the infection were observed in Bulgaria, Germany (Georgiev et al., 2013) and Iran (Nokhodian, Feizi, Ataei, Hoseini, & Mostafavi, 2017). Similarly, a study in Sweden (Ohlson et al., 2014) and a systematic review in Africa revealed that seroprevalence of *C. burnetii* infection is different, depending on the geographic location (Vanderburg et al., 2014). We believe that this heterogeneity could be due to differences in sample sizes among prefectures as well as due to agricultural and climatic differences among the tested areas. Moreover, as previously (Georgiev et al., 2013) we found that seropositivity measured at the individual animal level was lower than herd's seropositivity. In each herd only a relatively low number of animals seroconverted following contact with *C. burnetii*. This result is somewhat surprising, given the known high rate of infectivity of *C. burnetii* in ruminant populations.

In Greece, as elsewhere in the world (Angelakis & Raoult, 2010; Eldin et al., 2017), we found evidence of widespread exposure to *C. burnetii* in domestic ruminant populations. Recently, we raised the question of the under‐diagnosis of *C. burnetii* infections in Greece possibly due to the lack of awareness by physicians. In that study, we found evidence of *C. burnetii* infection in humans throughout most Greek territories as also a new endemic *C. burnetii* genotype (Karageorgou et al., 2020). Domestic ruminants are considered the primary reservoir for *C. burnetii* and most Q fever outbreaks in humans are mostly associated with close contact between people and infected ruminants after the inhalation of aerosols by animals abortions contaminated with *C. burnetii*. In addition, there is a consensus on the key role played by wind in the transmission of *C. burnetii* between ruminant farms and from ruminants to humans (Van Der Hoek et al., 2011). Indeed, wind was identified, as a cause of occurrence of *C. burnetii* infection, by assuming the airborne dispersal of contaminated particles from infected farms to surrounding populations (Nusinovici, Frossling, Widgren, Beaudeau, & Lindberg, 2015). A higher animal density could also increase the risk of propagation by increasing the potential number of neighbouring sources of contamination (Nusinovici et al., 2014). Moreover, a high quantity of precipitation it seems that can decrease the quantity of dust in the air and thus the likelihood of transmission (Hogerwerf et al., 2012). In this context and prompted by the outbreak of Q fever in the Netherlands (Van Der Hoek et al., 2012), the control of the propagation of *C. burnetii* within and between ruminant herds is an important public health and animal health issue. We believe that the implementation of effective *C. burnetii* control measures in ruminants in Greece could consequently have positive consequences on human health. In addition, in herds with high animal densities in windy areas and high temperatures, the vaccination of animals in both infected and *C. burnetii* free herds may be a relevant control measure to limit zoonotic risk (Courcoul et al., 2011).

In conclusion, we provide evidence of *C. burnetii* in productive ruminants throughout many Greek territories. We raise the question of Q fever emergence in Greece, due to the risk of inhalation of the bacterium by people who entered the affected farms. In all reported human outbreaks, animal contact with infected animals has been a consistent feature and the most likely source of *C. burnetii* infection (Eldin et al., 2017). However, to this date there is insufficient information to enable early prediction of large Q fever outbreaks in Greece. Considering the importance of *C. burnetii* for public health, our data reflect the lack of awareness by veterinarians, physicians and competent authorities alike. We believe that much remains unclear for *C. burnetii* and there is a need to improve the means for early detection of risk of outbreaks in Greece, the effectiveness of veterinary control measures, and determine the best follow‐up strategy in territories with repeated outbreaks over several years.

## CONFLICT OF INTEREST

The authors declare no conflict of interest.

## AUTHOR CONTRIBUTION

**Dimitrios Vourvidis:** Data curation; Supervision; Writing‐original draft. **Anna Kyrma:** Data curation; Investigation; Methodology. **Maria Linou:** Data curation; Methodology. **Sophie Edouard:** Writing‐original draft. **Emmanouil Angelakis:** Formal analysis; Supervision; Validation; Writing‐original draft.

### PEER REVIEW

The peer review history for this article is available at https://publons.com/publon/10.1002/vms3.337.

## References

[vms3337-bib-0001] Angelakis, E. , Million, M. , D’Amato, F. , Rouli, L. , Richet, H. , Stein, A. , … Raoult, D. (2012). Q fever and pregnancy: Disease, prevention, and strain specificity. European Journal of Clinical Microbiology and Infectious Diseases, 32, 361–368. 10.1007/s10096-012-1750-3 23052984

[vms3337-bib-0002] Angelakis, E. , & Raoult, D. (2010). Q fever. Veterinary Microbiology, 140, 297–309. 10.1016/j.vetmic.2009.07.016 19875249

[vms3337-bib-0003] Arricau‐Bouvery, N. , & Rodolakis, A. (2005). Is Q fever an emerging or re‐emerging zoonosis? Veterinary Research, 36, 327–349. 15845229 10.1051/vetres:2005010

[vms3337-bib-0004] Chochlakis, D. , Santos, A. S. , Giadinis, N. D. , Papadopoulos, D. , Boubaris, L. , Kalaitzakis, E. , … Petridou, E. I. (2018). Genotyping of *Coxiella burnetii* in sheep and goat abortion samples. BMC Microbiology, 18, 204 10.1186/s12866-018-1353-y 30514233 PMC6280429

[vms3337-bib-0005] Courcoul, A. , Hogerwerf, L. , Klinkenberg, D. , Nielen, M. , Vergu, E. , & Beaudeau, F. (2011). Modelling effectiveness of herd level vaccination against Q fever in dairy cattle. Veterinary Research, 42, 68 10.1186/1297-9716-42-68 21605376 PMC3125226

[vms3337-bib-0006] Eldin, C. , Mélenotte, C. , Mediannikov, O. , Ghigo, E. , Million, M. , Edouard, S. , … Raoult, D. (2017). From Q fever to *Coxiella burnetii* Infection: A Paradigm Change. Clinical Microbiology Reviews, 30(1), 115–190. 10.1128/CMR.00045-16 27856520 PMC5217791

[vms3337-bib-0007] Enserink, M. (2010). Infectious diseases. Questions abound in Q‐fever explosion in the Netherlands. Science, 327, 266–267. 10.1126/science.327.5963.266-a 20075230

[vms3337-bib-0008] Filioussis, G. , Theodoridis, A. , Papadopoulos, D. , Gelasakis, A. , Vouraki, S. , Bramis, G. , & Arsenos, G. (2017). Serological prevalence of *Coxiella burnetii* in dairy goats and ewes diagnosed with adverse pregnancy outcomes in Greece. Annals of Agricultural and Environmental Medicine, 24, 702–705. 10.26444/aaem/80706 29284250

[vms3337-bib-0009] Georgiev, M. , Afonso, A. , Neubauer, H. , Needham, H. , Thiery, R. , Rodolakis, A. , … Van der Hoek, W. (2013). Q fever in humans and farm animals in four European countries, 1982 to 2010. Eurosurveillance, 18(8), 20407. 23449232

[vms3337-bib-0010] Hogerwerf, L. , Borlée, F. , Still, K. , Heederik, D. , van Rotterdam, B ., de Bruin, A ., … Wouters, I. M. (2012). Detection of *Coxiella burnetii* DNA in inhalable airborne dust samples from goat farms after mandatory culling. Applied and Environmental Microbiology, 78, 5410–5412. 10.1128/AEM.00677-12 22582072 PMC3416441

[vms3337-bib-0011] Karageorgou, I. , Kogerakis, N. , Labropoulou, S. , Hatzianastasiou, S. , Mentis, A. , Stavridis, G. , & Angelakis, E. (2020). Q fever endocarditis and a new genotype of *Coxiella burnetii*, Greece. Emerging Infectious Diseases, 10. 10.3201/eid2610.191616 PMC7510691 32946732

[vms3337-bib-0012] Kokkini, S. , Chochlakis, D. , Vranakis, I. , Angelakis, E. , Tselentis, Y. , Gikas, A. , & Psaroulaki, A. (2013). Antibody kinetics in serological indication of chronic Q fever: The Greek experience. International Journal of Infectious Diseases, 17, e977–e980. 10.1016/j.ijid.2013.04.010 23773241

[vms3337-bib-0013] Million, M. , Thuny, F. , Bardin, N. , Angelakis, E. , Edouard, S. , Bessis, S. , … Raoult, D. (2016). Antiphospholipid antibody syndrome with valvular vegetations in acute Q fever. Clinical Infectious Diseases, 62, 537–544. 10.1093/cid/civ956 26585519

[vms3337-bib-0014] Million, M. , Walter, G. , Thuny, F. , Habib, G. , & Raoult, D. (2013). Evolution from acute Q fever to endocarditis is associated with underlying valvulopathy and age and can be prevented by prolonged antibiotic treatment. Clinical Infectious Diseases, 57, 836–844. 10.1093/cid/cit419 23794723

[vms3337-bib-0015] Nokhodian, Z. , Feizi, A. , Ataei, B. , Hoseini, S. G. , & Mostafavi, E. (2017). Epidemiology of Q fever in Iran: A systematic review and meta‐analysis for estimating serological and molecular prevalence. Journal of Research in Medical Sciences, 22, 121. 29259632 10.4103/jrms.JRMS_586_17 PMC5721492

[vms3337-bib-0016] Nusinovici, S. , Frossling, J. , Widgren, S. , Beaudeau, F. , & Lindberg, A. (2015). Q fever infection in dairy cattle herds: Increased risk with high wind speed and low precipitation. Epidemiology and Infection, 143, 3316–3326. 10.1017/S0950268814003926 25783480 PMC4594051

[vms3337-bib-0017] Nusinovici, S. , Hoch, T. , Widgren, S. , Joly, A. , Lindberg, A. , & Beaudeau, F. (2014). Relative contributions of neighbourhood and animal movements to *Coxiella burnetii* infection in dairy cattle herds. Geospatial Health, 8(2), 471–477. 10.4081/gh.2014.36 24893024

[vms3337-bib-0018] Ohlson, A. , Malmsten, J. , Frössling, J. , Bölske, G. , Aspán, A. , Dalin, A.‐M. , & Lindberg, A. (2014). Surveys on *Coxiella burnetii* infections in Swedish cattle, sheep, goats and moose. Acta Veterinaria Scandinavica, 56, 39 10.1186/1751-0147-56-39 25007979 PMC4112654

[vms3337-bib-0019] Pandit, P. , Hoch, T. , Ezanno, P. , Beaudeau, F. , & Vergu, E. (2016). Spread of *Coxiella burnetii* between dairy cattle herds in an enzootic region: Modelling contributions of airborne transmission and trade. Veterinary Research, 47, 48 10.1186/s13567-016-0330-4 27048416 PMC4822316

[vms3337-bib-0020] Pape, M. , Bouzalas, E. G. , Koptopoulos, G. S. , Mandraveli, K. , Arvanitidou‐Vagiona, M. , Nikolaidis, P. , & Alexiou‐Daniel, S. T. (2009). The serological prevalence of *Coxiella burnetii* antibodies in sheep and goats in northern Greece. Clinical Microbiology & Infection, 15(Suppl 2), 146–147. 10.1111/j.1469-0691.2008.02159.x 19456805

[vms3337-bib-0021] Prudent, E. , Lepidi, H. , Angelakis, E. , & Raoult, D. (2018). Fluorescence in situ hybridization (FISH) and peptide nucleic acid probe‐based FISH for diagnosis of Q fever endocarditis and vascular infections. Journal of Clinical Microbiology, 56(9), e00542‐18. 29899006 10.1128/JCM.00542-18 PMC6113452

[vms3337-bib-0022] Tissot‐Dupont, H. , Amadei, M. A. , Nezri, M. , & Raoult, D. (2004). Wind in November, Q fever in December. Emerging Infectious Diseases, 10, 1264–1269. 10.3201/eid1007.030724 15324547 PMC3323349

[vms3337-bib-0023] Van Der Hoek, W ., Hogema, B. M. , Dijkstra, F. , Rietveld, A. , Wijkmans, C. J. , Schneeberger, P. M. , Zaaijer, H. L. (2012). Relation between Q fever notifications and *Coxiella burnetii* infections during the 2009 outbreak in The Netherlands. Eurosurveillance Weekly, 17, 20058. 22297100

[vms3337-bib-0024] Van Der Hoek, W ., Meekelenkamp, J. C. , Leenders, A. C. , Wijers, N. , Notermans, D. W. , & Hukkelhoven, C. W. (2011). Antibodies against *Coxiella burnetii* and pregnancy outcome during the 2007–2008 Q fever outbreaks in The Netherlands. BMC Infectious Diseases, 11, 44 10.1186/1471-2334-11-44 21314933 PMC3042933

[vms3337-bib-0025] Vanderburg, S. , Rubach, M. P. , Halliday, J. E. , Cleaveland, S. , Reddy, E. A. , & Crump, J. A. (2014). Epidemiology of *Coxiella burnetii* infection in Africa: A OneHealth systematic review. PLoS Neglected Tropical Diseases, 8, e2787 10.1371/journal.pntd.0002787 24722554 PMC3983093

